# Longitudinal Associations between Physical and Cognitive Performance among Community-Dwelling Older Adults

**DOI:** 10.1371/journal.pone.0122878

**Published:** 2015-04-13

**Authors:** Magdalena I. Tolea, John C. Morris, James E. Galvin

**Affiliations:** 1 Alzheimer’s Disease Research Center, Departments of Neurology, Psychiatry, and Population Health, New York University School of Medicine, New York, NY, United States of America; 2 Charles F. and Joanne Knight Alzheimer’s Disease Research Center, Washington University, St. Louis, MO, United States of America; University Of São Paulo, BRAZIL

## Abstract

To assess the directionality of the association between physical and cognitive decline in later life, we compared patterns of decline in performance across groups defined by baseline presence of cognitive and/or physical impairment [none (n = 217); physical only (n = 169); cognitive only (n = 158), or both (n = 220)] in a large sample of participants in a cognitive aging study at the Knight Alzheimer’s Disease Research Center at Washington University in St. Louis who were followed for up to 8 years (3,079 observations). Rates of decline reached 20% for physical performance and varied across cognitive tests (global, memory, speed, executive function, and visuospatial skills). We found that physical decline was better predicted by baseline cognitive impairment (slope = -1.22, p<0.001), with baseline physical impairment not contributing to further decline in physical performance (slope = -0.25, p = 0.294). In turn, baseline physical impairment was only marginally associated with rate of cognitive decline across various cognitive domains. The cognitive-functional association is likely to operate in the direction of cognitive impairment to physical decline although physical impairment may also play a role in cognitive decline/dementia. Interventions to prevent further functional decline and development of disability and complete dependence may benefit if targeted to individuals with cognitive impairment who are at increased risk.

## Introduction

More than 5 mil Americans aged 65 years or older currently have Alzheimer’s disease (AD), with this figure expected to inflate to13.8 mil by 2050 [1]. Also, 23.7 mil (62%) of seniors report limitations in activities of daily living (ADL) [2], rendering dementia and disability important public health concerns, especially in the context of their frequent co-existence [3]. Associations between physical performance and individual cognitive domains (e.g. cognitive speed) have been reported [4] and cross-sectional analyses support an effect of physical performance across different cognitive subdomains [5]. Although associations between cognitive and physical function are strongest at later stages of dementia, there is increasing evidence that physical and cognitive factors interact at the earliest detectable stages of cognitive impairment [6], suggesting that early intervention holds promise of mitigating future decline.

However, the direction in which this association operates is still debated. In a recent survival analysis, risk of incident AD was increased in those with poor baseline physical performance [7], although no relationship [8] or inconsistent associations going from physical to cognitive functionality [9] have also been reported. The bulk of evidence suggests that the direction of the relationship proceeds from cognitive to functional decline. Poor performance on global and domain-specific measures of cognition, even when within the normal range for cognition [10], is associated with higher rates of physical decline, increased risk of ADL limitations, and early development of physical disability [10, 11].

Most previous work however has concentrated on testing the relationship between cognitive and physical function in a single direction [7,11–13] or assessed the co-existence of decline in both cognitive and physical function [14]. An alternative approach to better understanding the directionality between cognitive and physical function would be to assess the impact of baseline cognitive and/or physical function on the rates of cognitive and functional decline in a large sample of well-characterized older adults, with the expectation that the effect of baseline cognitive performance on physical decline would be greater than the impact of baseline physical performance on cognitive decline.

## Materials and Methods

### Study Participants

All participants were volunteers who enrolled in a longitudinal study of memory and aging at the Knight Alzheimer’s Disease Research Center (ADRC) at Washington University in St. Louis, Missouri initiated in 1979 and over the past 30 years evaluating over 3500 individuals. Participants are recruited via word of mouth, public service announcements, and referrals from physicians in the greater St. Louis metropolitan area. The ADRC enrolls community-dwelling older adults ranging from normal cognition to dementia who are assessed annually for cognitive and physical functionality. All participants undergo identical assessments and measures included in this study have been present throughout the longitudinal project. From this pool of ADRC participants, we selected individuals aged 50 years or older who were able to ambulate, completed clinical, cognitive, and physical assessments, and had ≥1 year of follow-up between January 1998 and December 2007. These dates coincide with the completion of one the primary measurements for this study—the Physical Performance Test. The characteristics of this sample are consistent with those of the entire cohort. Written informed consent was obtained from all study participants and the study protocol was approved by the Institutional Review Board at Washington University School of Medicine.

### Clinical and Cognitive Assessments

Participants received a detailed neurological examination and were administered the Mini Mental Status Examination (MMSE), the Short Blessed Test [15], and an aphasia battery. Dementia was diagnosed using standard criteria [16–19] and its severity was staged using the Clinical Dementia Rating (CDR) [20]. For the purpose of this study, cognitive impairment was defined as a CDR≥0.5 with a score of 0 indicating normal cognition. A further measure of global cognitive function was computed using the CDR sum of boxes (CDR-SB) by summing the six individual domain scores with a range of 0–18 [21]. A larger CDR-SB score indicates greater severity of impairment.

As part of the annual clinic visit, participants underwent extensive neuropsychological testing. Selected cognitive tests were used in the analysis to represent individual cognitive domains including verbal fluency measured with Animal Naming, semantic memory with Boston naming test (BNT), psychomotor speed with Trail Making Part A (TMA), and executive function with Trail Making Part B (TMB). Additional measurements of cognitive functioning associated with the ***Frontal*** (i.e. executive function), ***Temporal*** (i.e. memory/verbal), and ***Parietal*** (i.e. visuospatial) lobes as indexed by Kanne and colleagues [22] were also assessed corresponding to autopsy-confirmed distribution of AD pathology. A composite factor score (z-score) was derived for each person using weights obtained from a previous principal components analysis of all individuals without dementia enrolled in the ADRC longitudinal study [23]. The z-score was used as a ***Global*** measure of cognition in the current data analysis.

### Physical Performance

Physical performance on a modified 9-item Physical Performance Test (PPT), which was administered by a trained research nurse, was assessed at baseline and subsequent annual clinic visits. The original PPT includes 7 tasks: writing a sentence, simulating eating, lifting a book, simulating dressing, picking up a penny from the floor, turning in a complete circle, and walking 50 feet [23]. A 5-times sit-to-stand test and a progressive Romberg test were added as measures of lower extremity muscle strength and balance, known risk factors for falling [24], institutionalization [25], and mortality [26]. Each of the 9 tasks was scored on a 5-point scale (0 = lowest through 4 = highest performance). A total PPT score was computed by summing up these items (range 0–36) with lower scores indicating poor functionality. Physical impairment was defined as a total PPT<28 with scores of ≥28 indicative of normal physical functionality [27].

### Data analysis

Based on their baseline global CDR and total PPT score, participants were categorized into cognitively (CDR = 0) and physically (PPT≥28) normal (not impaired–**NI**); physically impaired (PPT<28) but cognitively normal (CDR = 0) (**PI**); cognitively impaired (CDR≥0.5) but physically normal (PPT≥28) (**CI**); or cognitively and physically impaired (CDR≥0.5, PPT<28) (**CPI**). Overall mean and proportion differences in socio-demographic and other significant factors between the four different impairment groups were tested with analysis of variance for continuous variables or chi square tests for categorical variables (**Table 1**).

**Table 1:** Baseline characteristics according to impairment status.

	NI	PI	CI	CPI
N	217	169	158	220
Age, yrs.	73.76±7.66	80.56±9.00	73.49±7.13	79.19±7.92
% female	56.2	69.2	44.3	59.6
% white	94.5	88.2	92.4	86.4
Education, yrs.	14.84±2.83	14.37±3.13	14.39±3.07	13.10±3.18
Total PPT	30.53±1.75	22.18±6.37	30.09±1.87	21.71±5.88
PPT—gait items	20.69±1.39	14.49±4.70	20.45±1.57	14.51±4.26
PPT—non-gait items	9.84±1.16	7.69±2.21	9.65±1.11	7.20±2.29
CDR-SB	0.04±0.14	0.07±0.1	3.11±1.75	3.82±2.58
TMA	36.58±13.40	46.23±18.60	54.41±31.37	74.56±37.25
TMB	91.37±35.98	119.45±39.41	133.52±47.21	158.08±33.69
Word fluency	31.15±10.22	28.20±11.07	23.37±10.74	18.73±8.99
BNT	55.64±4.81	52.51±6.18	47.06±10.19	41.54±13.40
Composite cognitive score	0.33±0.88	-0.41±0.96	-1.45±1.44	-2.39±1.51
Frontal factor score	0.59±0.66	0.32±0.68	0.07±0.77	-0.26±0.76
Temporal factor score	1.41±0.60	1.06±0.69	0.12±0.81	-0.20±0.82
Parietal factor score	1.00±0.53	0.56±0.52	0.33±0.75	-0.24±0.78

**Notes:** NI = not impaired, PI = physically impaired but cognitively normal, CI = cognitively impaired but physically normal, CPI = cognitively and physically impaired; PPT = Physical Performance Test; CDR-SB = CDR Sum of boxes; TMA = Trail making part A; TMB = Trail making part B; BNT = Boston Naming test; All (overall) differences are statistically significant at p<0.001, with the exception of race which was significant at p<0.05. Individuals who were NI at baseline tended to perform best while individuals who were CPI at baseline tended to perform the worst.

Mixed-effects regression models were used to assess longitudinal associations between baseline impairment category (described above) and declines in physical performance (PPT score) and cognitive performance, the latter measured globally (i.e. CDR-SB, composite factor score) and at the domain level (i.e. (Animal naming, BNT, TMA, TMB, the 3 Kanne factors). The main term for impairment status was interpreted as indicating differences in outcome level between impairment groups at baseline, and interaction terms with time (i.e. impairment group X time) as indicating differences in the rate of decline in physical or cognitive functionality between impairment groups as defined at baseline. An initial unadjusted model (Model 1) was compared against models adjusted for age, gender, education, race (Model 2), and additionally for baseline functional performance (Model 3) to determine the impact of significant risk factors on the effect of baseline impairment status (**Tables 2 and 3**).

**Table 2:** Rate of decline in physical performance by baseline impairment status.

	Model 1		Model 2		Model 3	
	Slope	P value	Slope	P value	Slope	P value
**Intercept**	30.32	<0.001	46.70	<0.001	10.40	<0.001
**Overall decline**	-0.76	<0.001	-0.50	<0.001	-0.70	<0.001
**Baseline impairment**						
NI	Ref		Ref		Ref	
PI	-7.25	<0.001	-5.59	<0.001	0.04	0.912
CI	-1.07	0.036	-1.16	0.021	-0.58	0.138
CPI	-8.42	<0.001	-6.92	<0.001	-0.71	0.102
Baseline impairment*Time						
NI * Time	Ref		Ref		Ref	
PI * Time	-0.29	0.164	-0.28	0.158	-0.25	0.294
CI * Time	-1.03	<0.001	-0.98	<0.001	-1.22	<0.001
CPI * Time	-1.09	<0.001	-1.04	<0.001	-1.17	<0.001

**Notes**: baseline impairment status is based on baseline PPT score (score of <28 being used as indication of physical impairment). NI = not impaired, PI = physically impaired but cognitively normal, CI = cognitively impaired but physically normal; CPI = cognitively and physically impaired; Performance on the PPT (score) was also used to measure decline in physical function; Model 1 = unadjusted; Model 2 = Model 1 + age + gender + education + race; Model 3 = Model 2 + baseline total PPT score; Rates of decline in physical performance were higher in the CI and CPI groups than in the NI (reference) group. No significant differences were observed in the PI group compared with the NI group.

**Table 3:** Rate of decline in cognitive performance by baseline impairment status (full models shown).

	Intercept	Overall decline	Baseline effects				Longitudinal effects			
			NI	PI	CI	CPI	NI	PI	CI	CPI
**CDR-SB**	0.06 (0.847)^£^	0.10 0.166)	Ref	-0.01 (0.864)	-0.18 (0.059)	-0.14 (0.138)	Ref	0.15 (0.176)	1.33 (<0.001)	1.65 <0.001)
**Word fluency**	3.03 (0.034)	0.08 0.577)	Ref	-0.28 (0.439)	-0.89 (0.020)	-1.23 (0.001)	Ref	-0.25 (0.280)	-1.76 (<0.001)	-1.82 (<0.001)
**BNT**	0.02 (0.985)	-0.22 (0.267)	Ref	0.15 (0.569)	0.31 (0.257)	-0.05 (0.867)	Ref	-0.35 (0.251)	-2.62 (<0.001)	-2.93 (<0.001)
**TMA**	-1.38 (0.784)	0.52 (0.479)	Ref	0.71 (0.563)	0.32 (0.799)	3.11 (0.020)	Ref	1.47 (0.199)	10.59 (<0.001)	11.75 (<0.001)
**TMB**	-1.01 (0.872)	1.79 (0.002)	Ref	3.06 (0.047)	6.30 (<0.001)	8.97 (<0.001)	Ref	0.08 (0.932)	5.73 (<0.001)	2.87 (0.008)
**Composite cognition score**	0.14 (0.266)	-0.01 (0.645)	Ref	-0.01 (0.706)	-0.01 (0.836)	-0.05 (0.214)	Ref	-0.07 (0.080)	-0.45 (<0.001)	-0.45 (<0.001)
**Frontal factor score**	0.10 (0.351)	0.02 (0.054)	Ref	-0.02 (0.538)	-0.03 (0.356)	-0.09 (0.003)	Ref	-0.02 (0.261)	-0.14 (<0.001)	-0.14 (<0.001)
**Temporal factor score**	0.15 (0.059)	-0.01 (0.646)	Ref	-0.04 (0.050)	-0.09 (<0.001)	-0.11 (<0.001)	Ref	-0.02 (0.209)	-0.15 (<0.001)	-0.15 (<0.001)
**Parietal factor score**	-0.02 (0.773)	0.02 (0.067)	Ref	-0.01 (0.524)	0.02 (0.337)	-0.04 (0.026)	Ref	-0.03 (0.107)	-0.15 (<0.001)	-0.17 (<0.001)

^£^ Slope (p value); NI = not impaired, PI = physically impaired but cognitively normal, CI = cognitively impaired but physically normal, CPI = cognitively and physically impaired; PPT = Physical Performance Test; CDR-SB = CDR Sum of boxes; TMA = Trail making part A; TMB = Trail making part B; BNT = Boston Naming test; Baseline physical impairment status is based on baseline total PPT (<28). Baseline cognitive impairment was based on CDR score (≥0.5). Cognitive performance was assessed by several psychometric tests including CDR-SB, Animal testing, TMA, TMB, BNT and Factor scores.

Models are adjusted for age, gender, education, race, and baseline cognitive performance. Rates of decline in cognitive performance were higher in the CI and CPI groups than in the NI (reference) group. No significant differences were observed in the PI group compared to the NI group.

In addition, given that a number of the physical performance tasks assessed by the PPT are gait/balance related (i.e. lifting a book, picking up penny, turning in a complete circle, walking for 50 feet, chair raising, and balance; range 0–24) and could therefore impact our results, we dichotomized gait/balance items from those items that are not gait/balance-related (i.e. writing a sentence, simulating eating, and simulating dressing; range 0–12). Because no validated cutoff points are available for these sub-scales, we used the median (19 for the gait items and 9 for the non-gait items) to be consistent with the published cutoff for the PPT scale [27], which represented the median score in our sample. These cutoff points were used to reconstruct the baseline impairment status (our predictor variable) to reflect these 2 different physical performance components (gait/balance vs. non-gait) and to assess them in relation to decline in physical (**S1 Table**) and cognitive performance (**S2 Table**). A p<0.05 was used to indicate statistical significance. Analyses were performed in SAS 9.3 (SAS Institute Inc., Cary, NC).

## Results

A total number of 764 participants in the ADRC longitudinal study were eligible for participation in this study and were therefore included in the current analysis for a total of 3,079 observations. Follow-up time ranged from 1–8 years (mean = 2.6±1.6yrs). Twenty eight percent of our participants were non-impaired (NI), 22.1% were physically impaired only (PI), 20.7% cognitively impaired only (CI), and 28.8% dually impaired at baseline (CPI). Other significant baseline differences were observed, with the PI and CPI groups being older, more likely to belong to a minority group, and to be less educated. **Table 1**presents the mean scores for physical functionality, global cognition, processing speed, executive function, and language. As expected, NI individuals had the best scores across all domains, while CPI individuals had the worst scores.

**Fig 1:**
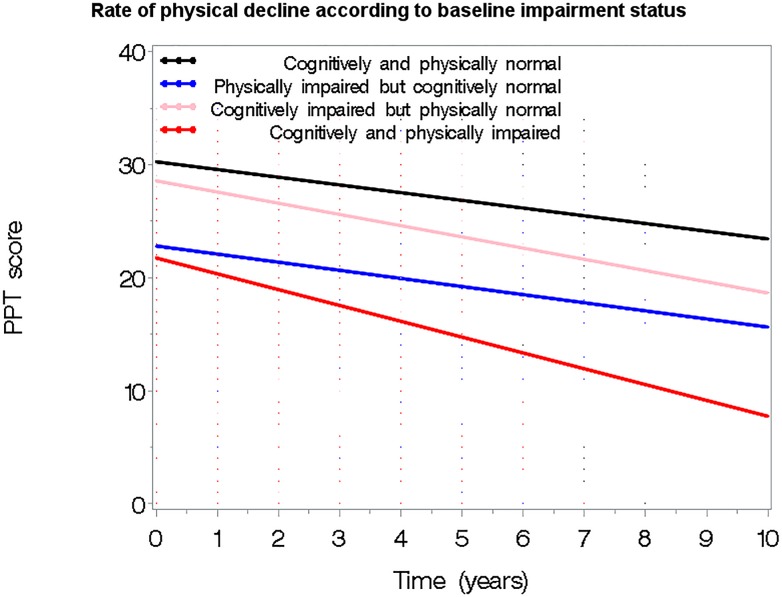
Rate of physical functional decline based on baseline cognitive/physical impairment status. Significantly steeper decline in physical performance (measured with PPT score) was noted in the CI and CPI groups, although physical impairment does not add much above what is seen in the CI group. In contrast, the PI group appears to decline in physical functionality at similar rates with the NI. Results are from the full model adjusted for age, gender, education, race and baseline physical performance.

The average rate of decline was 17% for PPT, 25.4% for TMA, 13.9% for TMB, 5.6% for word fluency, 7.5% for BNT, and 168% for CDR-SB, 22.2% for the Frontal factor, 81.7% for the Temporal factor, and 28.2% for the Parietal factor. Significant differences in rates of change between the 4 groups were found with the CPI group showing the highest rate of decline. For example, the mean rate of decline (i.e. proportion decline from baseline to the last follow-up) in CDR-SB was 0.27 (±0.28) for NI; 0.30 (±0.25) for PI; 1.43 (±0.10) for CI, and 1.82 (±0.10) for the CPI group. Findings were similar for the other cognitive measures studied. In mixed effects models, the rate of decline in physical performance was dependent on presence of cognitive impairment at baseline (**Table 2, Model 1**). Compared to the NI group, the CI group declined by a rate of 1.03 (p<0.001) and the CPI group by a rate of 1.09 (p<0.001) while PI participants were similar to the NI group (estimate = -0.29, p = 0.164). Adjustment for age, gender, education, race, and baseline total PPT score (**Table 2, Models 2 and 3**) did not significantly impact the effect of baseline impairment status; both CI and CPI groups continued to show a significantly greater rate of decline in their PPT score compared to the NI group. Accounting for all factors, the overall rate of decline in PPT was 0.70 units per year (p<0.001). These associations are graphically presented in **Fig 1**.

**Fig 2:**
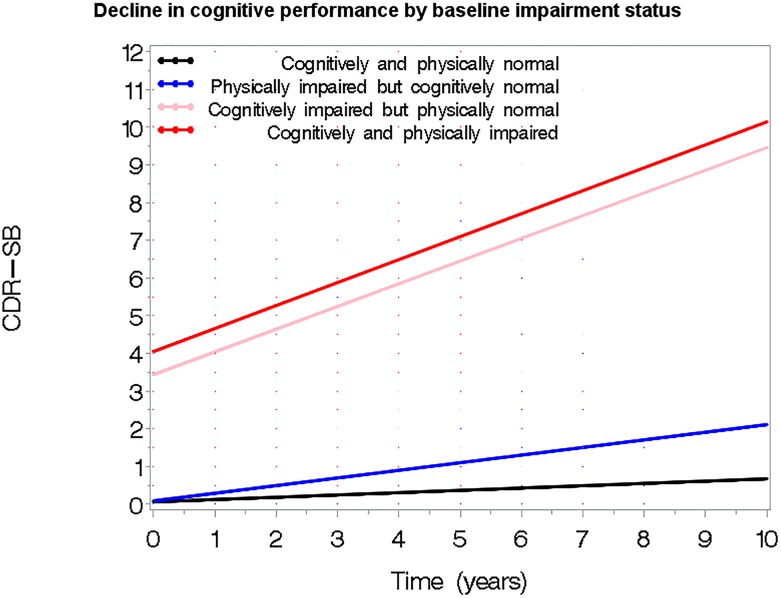
Decline in cognitive function by baseline cognitive/physical impairment status. Significantly steeper decline in global cognitive performance (measured with CDR-SB score) was noted in the CI and CPI groups, although physical impairment does not add much above what is seen in the CI group. In contrast, the PI group appears to decline in physical functionality at similar rates with the NI. Results are from the full model adjusted for age, gender, education, race and baseline physical performance.

We next assessed the effect of baseline impairment status on decline in individual cognitive measures. Fully adjusted models predicting decline in Global z-scores, TMA, TMB, word fluency, BNT, CDR-SB, and the 3 domain factors (Frontal, Temporal, Parietal) are presented in **Table 3**. The significant interaction terms with time suggest that while the PI group initially appears to decline faster than the NI group, the difference was only marginally significant in the composite cognition measures. In contrast, the CI and CPI groups have a sharper rate of decline across all cognitive measures investigated in this study. For example, being CI was associated with a 1.76 greater rate of decline in word fluency (p>0.001) and being CPI resulted in a rate of decline that was 1.82 greater (p<0.001) than being non-impaired. Similarly, the rate of decline in CDR-SB (higher scores over time) was higher in the CI group (estimate = 1.33, p<0.001) and highest in the CPI group (estimate = 1.65, p<0.001) (**Fig 2**). These results persisted when we looked separately at the gait vs. non-gait components of PPT (**S1 and S2 Tables**).

## Discussion

In a large sample of well-characterized, community-dwelling older adults, we investigated the longitudinal association between cognitive and physical impairment with a special focus on the directionality of this association. Using mixed-effects regression models to predict decline in both cognitive and physical aspects of functionality based on presence of cognitive impairment, physical impairment, or both at baseline, we found evidence that cognitive impairment is a stronger predictor of physical decline than physical impairment is a predictor of cognitive decline. More specifically, our results indicate that cognitive impairment is associated with steeper cognitive (global and individual domains) and physical decline, while physical impairment appears to have little impact of its own on either or on the effect of cognitive impairment.

Given the impact that cognitive and physical disability have on our aging population, an interest in the processes that lead to these common health conditions, and particularly whether each one plays a role in the development of the other may have important implications for health promotion and prevention studies. Theoretical models have been proposed to explain how and at what point cognitive or physical impairment may influence one another. The model proposed by Nagi—a well-known and utilized disability models among gerontologists—suggests that cognitive impairments may be implicated in the process leading to development of disability by increasing the risk of functional limitations (e.g. reduced mobility) which in turn may lead to impairment in the ability to perform ADLs (disability) [28]. Our results of a strong effect of cognitive impairment on rate of physical decline provide support for the hypothesized role of cognitive impairment as either a risk factor or exacerbator of physical decline. Age-related declines in cardiopulmonary, orthopedic, visual, proprioceptive, and vestibular systems may all play a role in loss of mobility [29]. Neurodegenerative processes associated with AD and related disorders may further exacerbate these declines in mobility and subsequent disability. In addition, other pathways in the cognition-functionality association may include chronic inflammation [30,31] metabolic syndrome [32,33] and stress [34,35].

Empirical evidence for an association between reduced cognitive abilities and functional decline and incident disability provides support for this proposed biological link [10,13,36]. For example, in a longitudinal study of 5,317 initially non-disabled community-dwelling older adults followed for up to 8 years, lower baseline cognitive function predicted an earlier age of onset of functional disability and an accelerated rate of progression once disability settled, while physical function did not have an impact on rate of disability progression [36]. In line with these findings, we found a steeper rate of physical decline in the groups that were cognitively impaired at baseline but not in those physically impaired. Moreover, not only did baseline physical performance status not predict further physical decline by itself but it also did not increase the rate of decline above the effect of cognitive impairment suggesting that early detection of cognitive impairment alone could be used as strategy to identify older adults at risk of functional decline despite their level of physical impairment. Using a performance-based measure of physical function, we were able to show that the effect of cognitive impairment on physical decline is robust, holding true not only when the individual is asked to report such decline but also when she/he is observed performing physical tasks, as well as across various cognitive measures. In addition, we were able to show that the effects of cognitive impairment on physical decline extend across differential aspects of physical functioning involving upper and lower extremity-related tasks. Moreover, by establishing global functionality (NI, CI, PI, CPI) at baseline, we were able to demonstrate the importance of baseline cognitive function for future physical limitations before the stage of disability (the outcome of preference for most previous reports) is reached., The reverse association is less clear. We did not find compelling support that baseline impaired physical performance drives a decline in cognitive abilities. While we have previously reported a higher risk of dementia in initially dementia-free community-dwelling older adults with mild physical impairment [7], physical impairment itself does not appear to be an important facilitator for cognitive decline, which is better predicted by presence of cognitive impairment. Moreover, as was the case with the physical decline outcome, not only does physical impairment not affect the rate of cognitive decline, but it also has no significantly impact on the effect on cognitive impairment, suggesting a lack of effect modification between physical and cognitive impairment.

Although in both studies we drew from the same pool of participants, our current sample was less restrictive allowing individuals with missing neuropsychological data to be retained into the study as long as they had valid physical performance and CDR data. This differed from our previous study in which, to be included, participants had to have valid data on all assessments. To rule out the possibility that the inclusion of missing cognitive data would explain the difference in findings, we reran our models predicting decline in individual cognitive measures in a subset of 742 participants with valid data on all clinical assessments (2,500 total observations), also excluding baseline cognition from the models to mirror analyses performed in our previous study. This sensitivity analysis supported our initial findings of marginally significant prediction by baseline physical impairment (the PI group) on the composite Global z-score—a more global measure. In addition, the rate of decline in word fluency and psychomotor speed was significantly impacted by presence of PI at baseline (estimate = -0.46, p = 0.032 for word fluency and estimate = 1.43, p = 0.042 for TMA) although the other cognitive measures remained unaffected. Inconsistent contributions of physical function measured by self-reported ADL limitations to decline in cognitive abilities have been reported, with physical function either having no impact on future cognitive performance [8] or predicting cognitive abilities in the short but not the long run [9]. Our findings of no effect of objectively measured physical impairment on future cognitive decline suggest that this lack of impact is consistent across objective and subjective measures of physical function.

It may be that physical impairment predicts decline in cognition and development of dementia but measurements of cognitive impairment (informant-based vs. performance-based) may lead to difference in outcomes. Alternatively, development of physical impairment rather than baseline impairment may be a better predictor of cognitive decline [37]. There is increasing evidence supporting biological mechanisms linking physical limitation to cognitive decline with greater physical functionality positively impacting level of physical activity, which in turn improves cerebral blood flow.[38] This is likely to result in better cognitive performance, help maintain cognitive function, and possibly prevent or delay cognitive decline [39]. Another consideration is that physical impairment, at least as relates to fall risk, may operate in the pre-clinical stage of AD [40] and have less influence once cognitive impairment is manifest.

Our study has limitations. Although consisting of community-dwelling older adults, our sample may not be representative of the general population on potentially important variables such as race and educational attainment. It is possible that important risk factors for cognitive and functional decline were not assessed in our study. For example, levels of physical activity—an important risk factor for functional and cognitive decline was not available, potentially leading to overestimation of observed effects. In addition, the average follow-up time was less than 3 years, which may be insufficient to detect significant change in cognitive performance. However, while this was true for most of the individual cognitive domains assessed in our study, a significant decline in global cognition was observed over the study period, suggesting that this limitation may not have played a large role in the null effects of baseline physical impairment observed in this study. This argument is further weakened by the significant impact of baseline cognitive impairment on rate of decline in all measures of cognition investigated.

These limitations are offset by the large well-characterized, identically-assessed sample, the analytic technique used to predict decline in functionality, the use of established, validated objective measures of cognitive and physical performance, and the method of assessing the directionality of the association between cognitive and functional impairment. Although other studies have investigated the bi-directionality of this association and found it to operate from cognitive to physical impairment [8], the measure used in our study to assess presence and timeliness of baseline cognitive and physical impairment is unique. Another advantage relates to the concomitant investigation of the cognitive-to-physical and physical-to-cognitive relationships in the same cohort over a relatively large period of time.

## Conclusions

We found that the cognitive-functional association is likely to largely operate in the direction of cognitive impairment to physical decline, although a role of physical impairment in the process that leads to dementia is also possible and should continue to be probed. This directionality has been previously evidenced in middle-aged adults [8]. By extending these findings to later life, our study suggests that interventions to preserve functionality and prevent decline although best applied earlier in life, could also be effective later in life, particularly among individuals with cognitive impairment who are at increased risk of functional decline. This increased risk is not moderated by physical impairment status, suggesting that preventive interventions could be effective in cognitively impaired older adults regardless of their functional status. With an increasing interest in non-pharmacological approaches to prevent dementia (e.g., exercise programs, cognitive stimulation, social engagement, dietary approaches) our data suggests that early interventions in declining physical and cognitive abilities could mitigate risk of future dementia. Understanding the role of cognitive impairment in the process that leads to functional decline may also provide important information when forecasting needs for services in this vulnerable population.

## Supporting Information

S1 Table Rate of physical decline by baseline impairment status based on gait vs. non-gait components (full models). Models are adjusted for age, gender, education, race, and baseline gait/non-gait performance; Baseline physically impairment based on gait/balance and non-gait items depending on what component of physical performance was assessed longitudinally; NI = not impaired, PI = physically impaired, CI = cognitively impaired, CPI = cognitively and physically impaired; Gait/balance items: lifting a book, picking up penny, turning in a complete circle, walking for 50ft, chair raises, and the Romberg balance test; Non-gait items: writing a sentence, simulating eating, and simulating dressing. Rates of decline in physical performance (both gait/balance and non-gait items) were higher in the CI and CPI groups than in the NI (reference) group. No significant differences were observed in the PI group.
(DOCX)

S2 Table Rate of decline in specific cognitive functions by baseline impairment. status. Only the longitudinal effects from fully adjusted models are shown; NI = not impaired, PI = physically impaired, CI = cognitively impaired, CPI = cognitively and physically impaired; CDR-SB = CDR-sum of boxes; BNT = Boston naming test; TMA = Trail making part A; TMB = Trial making part B. Models are adjusted for age, gender, education, race, and baseline cognitive performance. Rates of decline in cognitive performance were higher in the CI and CPI groups than in the NI (reference) group. No significant differences were observed in the PI group.
(DOCX)
