# Supplementary material for: Longitudinal Associations between Physical and Cognitive Performance among Community-Dwelling Older Adults
Source: PLoS One. 2015 Apr 13;10(4):e0122878. doi: 10.1371/journal.pone.0122878 (PMC4395358; doi:10.1371/journal.pone.0122878)
Supplement: S1 Table — Models are adjusted for age, gender, education, race, and baseline gait/non-gait performance; Baseline physically impairment based on gait/balance and non-gait items depending on what component of physical performance was assessed longitudinally; NI = not impaired, PI = physically impaired, CI = cognitively impaired, CPI = cognitively and physically impaired; Gait/balance items: lifting a book, picking up penny, turning in a complete circle, walking for 50ft, chair raises, and the Romberg balance test; Non-gait items: writing a sentence, simulating eating, and simulating dressing. Rates of decline in physical performance (both gait/balance and non-gait items) were higher in the CI and CPI groups than in the NI (reference) group. No significant differences were observed in the PI group. (DOCX) [file pone.0122878.s001.docx]

**S1 Table. Rate of physical decline by baseline impairment status based on gait vs. non-gait components (full models).**

|  | **Gait/balance items** | | **Non-gait items** | |
| --- | --- | --- | --- | --- |
|  | Slope | P value | Slope | P value |
| **Intercept** | 8.842 | <0.001 | 3.178 | <0.001 |
| **Overall decline** | -0.537 | <0.001 | -0.186 | <0.001 |
| **Baseline effects** |  |  |  |  |
| NI | Ref |  | Ref |  |
| PI | 0.215 | 0.503 | -0.065 | 0.399 |
| CI | -0.372 | 0.190 | -0.157 | 0.205 |
| CPI | -0.244 | 0.439 | -0.664 | <0.001 |
| **Longitudinal effects** |  |  |  |  |
| NI | Ref |  | Ref |  |
| PI | -0.111 | 0.505 | -0.005 | 0.947 |
| CI | -0.690 | <0.001 | -0.475 | <0.001 |
| CPI | -0.617 | <0.001 | -0.378 | <0.001 |

**Notes**: Models are adjusted for age, gender, education, race, and baseline gait/non-gait performance; Baseline physically impairment based on gait/balance and non-gait items depending on what component of physical performance was assessed longitudinally; NI=not impaired, PI=physically impaired, CI=cognitively impaired, CPI=cognitively and physically impaired; Gait/balance items: lifting a book, picking up penny, turning in a complete circle, walking for 50ft, chair raises, and the Romberg balance test; Non-gait items: writing a sentence, simulating eating, and simulating dressing. Rates of decline in physical performance (both gait/balance and non-gait items) were higher in the CI and CPI groups than in the NI (reference) group. No significant differences were observed in the PI group.
