# Supplementary material for: Longitudinal Associations between Physical and Cognitive Performance among Community-Dwelling Older Adults
Source: PLoS One. 2015 Apr 13;10(4):e0122878. doi: 10.1371/journal.pone.0122878 (PMC4395358; doi:10.1371/journal.pone.0122878)
Supplement: S2 Table — Only the longitudinal effects from fully adjusted models are shown; NI = not impaired, PI = physically impaired, CI = cognitively impaired, CPI = cognitively and physically impaired; CDR-SB = CDR-sum of boxes; BNT = Boston naming test; TMA = Trail making part A; TMB = Trial making part B. Models are adjusted for age, gender, education, race, and baseline cognitive performance. Rates of decline in cognitive performance were higher in the CI and CPI groups than in the NI (reference) group. No significant differences were observed in the PI group. (DOCX) [file pone.0122878.s002.docx]

**S2 Table. Rate of decline in specific cognitive functions by baseline impairment status.**

|  | **Gait/balance component of physical performance** | | | | **Non-gait component of physical performance** | | | |
| --- | --- | --- | --- | --- | --- | --- | --- | --- |
|  | **NI** | **PI** | **CI** | **CPI** | **NI** | **PI** | **CI** | **CPI** |
|  |  | Slope  (p value) | Slope  (p value) | Slope  (p value) |  | Slope  (p value) | Slope  (p value) | Slope  (p value) |
| **CDR-SB** | Ref | 0.142  (0.198) | 1.323  (<0.001) | 1.645  (<0.001) | Ref | 0.087  (0.452) | 1.254  (<0.001) | 1.753  (<0.001) |
| **Word fluency** | Ref | -0.268  (0.256) | -1.668  (<0.001) | -1.919  (<0.001) | Ref | -0.450  (0.065) | -1.601  (<0.001) | -2.179  (<0.001) |
| **BNT** | Ref | -0.387  (0.201) | -2.614  (<0.001) | -2.960  (<0.001) | Ref | -0.431  (0.171) | -2.481  (<0.001) | -3.204  (<0.001) |
| **TMA** | Ref | 1.149  (0.317) | 11.290  (<0.001) | 10.939  (<0.001) | Ref | 0.872  (0.456) | 8.409  (<0.001) | 14.150  (<0.001) |
| **TMB** | Ref | 0.199  (0.829) | 5.273  (<0.001) | 3.324  (<0.001) | Ref | 0.546  (0.568) | 5.295  (<0.001) | 2.796  (0.020) |
| **Composite cognition score** | Ref | -0.068  (0.071) | -0.460  (<0.001) | -0.443  (<0.001) | Ref | -0.037  (0.341) | -0.378  (<0.001) | -0.516  (<0.001) |
| **Kanne frontal factor** | Ref | -0.021  (0.226) | -0.131  (<0.001) | -0.147  (<0.001) | Ref | -0.028  0.113) | -0.131  (<0.001) | -0.156  (<0.001) |
| **Kanne temporal factor** | Ref | -0.023  (0.142) | -0.138  (<0.001) | -0.162  (<0.001) | Ref | -0.024  (0.132) | -0.138  (<0.001) | -0.166  (<0.001) |
| **Kanne parietal factor** | Ref | -0.029  (0.084) | -0.160  (<0.001) | -0.162  (<0.001) | Ref | -0.024  (0.176) | -0.131  (<0.001) | -0.202  (<0.001) |

**Notes**: Only the longitudinal effects from fully adjusted models are shown; NI=not impaired, PI=physically impaired, CI=cognitively impaired, CPI=cognitively and physically impaired; CDR-SB=CDR-sum of boxes; BNT=Boston naming test; TMA=Trail making part A; TMB=Trial making part B. Models are adjusted for age, gender, education, race, and baseline cognitive performance. Rates of decline in cognitive performance were higher in the CI and CPI groups than in the NI (reference) group. No significant differences were observed in the PI group.
